# LncRNA MCM3AP-AS1 is downregulated in atherosclerosis and sponges miR-448 to suppress vascular smooth muscle cell proliferation

**DOI:** 10.1097/MD.0000000000033731

**Published:** 2023-06-02

**Authors:** Shiping Liu, Xiaoyi Qin

**Affiliations:** a Department of Geriatric Medicine, Ganzhou People’s Hospital, No.17, Hongqi Avenue, Zhanggong District, Ganzhou City, Jiangxi Province 341000, P. R. China.

**Keywords:** atherosclerosis, MCM3AP-AS1, MEF2C, miR-448

## Abstract

Long noncoding RNA MCM3AP-AS1 plays critical roles in cancers, but its role in atherosclerosis is yet to be elucidated. The expression of MCM3AP-AS1 in atherosclerosis and control plasma samples were measured by RT-qPCR. IntaRNA was used to predict potential base pairings between MCM3AP-AS1 and miR-448, and the results were confirmed by a dual luciferase activity assay. Cell proliferation assay was performed to explore the role of overexpression of MCM3AP-AS1, miR-448, and myocyte enhancer factor 2 (MEF2)-C in the proliferation of human aortic smooth muscle cells (HAOSMCs). MCM3AP-AS1 was downregulated in atherosclerosis and directly interacted with miR-448, which is a critical player in the proliferation of HAOSMCs, indicating its involvement in atherosclerosis. However, MCM3AP-AS1 and miR-448 showed no role in regulating the expression of each other. In contrast, overexpression of MCM3AP-AS1 increased the expression levels of MEF2-C, which can be targeted by miR-448. Moreover, MCM3AP-AS1 was found to inhibit the effects of miR-448 overexpression on both HAOSMC proliferation and MEF2-C expression. MCM3AP-AS1 is downregulated in atherosclerosis and sponges miR-448 to suppress the proliferation of HAOSMCs.

## 1. Introduction

High cholesterol, smoking and high blood pressure may cause endothelium damages.^[1]^ The damaged endothelium may result in the development of arterial plaque, which in turn can lead to inadequate delivery of oxygen-rich blood to heart and other parts of the body and subsequent atherosclerosis,^[2]^ the leading cause of coronary artery diseases and high mortality rate.^[3]^ At early stage, atherosclerosis is mostly reversible and can be cured with cholesterol-lowering therapy.^[4]^ However, atherosclerosis becomes irreversible at advanced stage and cholesterol-lowering therapy is not effective.^[5]^ Therefore, early diagnosis and effective treatment of atherosclerosis is crucial.

The molecular mechanism of atherosclerosis involves multiple pathways at the molecular level.^[6,7]^ Functional analyses have shown that some factors can be targeted to improve atherosclerosis therapy.^[8]^ It is known that long noncoding RNAs (lncRNAs) and micro RNAs (miRNAs) are involved in various human diseases including atherosclerosis.^[9,10]^ Although both of lncRNAs and miRNAs do not encode proteins, both can influence protein synthesis and are able to regulate proliferation, migration and matrix synthesis of vascular smooth muscle cells (VSMCs), which participate in different aspects of the formation of atherosclerotic lesion.^[11]^ The excessive growth of VSMCs can lead to the development of plaques, however, in later stages of plaque development, VSMCs can play a crucial role in preventing the fibrous cap from rupturing, thereby providing a beneficial effect.^[11]^ However, the functions of most lncRNAs and miRNAs in atherosclerosis remain unknown. It has been reported that miR-448 could target myocyte enhancer factor 2 (MEF2) to promote the proliferation of VSMC.^[12]^ Our bioinformatic analysis revealed that miR-448 was predicted to interact with MCM3AP-AS1, an oncogenic lncRNA that has been implicated in several types of cancer.^[13–16]^ We hypothesized that MCM3AP-AS1 may interact with miR-448, thereby indirectly regulating the expression of MEF2. Therefore, we sought to investigate the potential crosstalk among miR-448, MCM3AP-AS1 and MEF2 in atherosclerosis.

## 2. Methods

### 2.1. Atherosclerosis patients and healthy controls

This study was approved by the Ethic Committee of Ganzhou people’s Hospital, China. A total of 60 patients diagnosed with atherosclerosis (34 males and 26 females, with a mean age of 33.1 ± 3.5 years and an age range of 26–39 years) and 60 heathy controls (34 males and 26 females, with a mean age of 33.2 ± 3.4 years and an age range of 26–39 years) who were admitted to the Ganzhou people’s Hospital of Shandong University between July 2017 and July 2019 were enrolled in this study. Written informed consent was obtained from all patients prior to their participation in the study. Of the 60 patients, 27, 20, and 13 were classified at pathological stage I, II, and III, respectively, based on the plaque formation. And 12, 19, 17, and 14 patients were classified at step 2, 3, 4, and 5, respectively, based on the development of atherosclerosis. The clinical features of atherosclerosis patients and controls were shown in Table 1.

**Table 1:** Clinical features of patients and control.

Features	Atherosclerosis (n = 60)	Control (n = 60)
Age (yr)	33.1 ± 3.5	33.2 ± 3.4
Gender		
Male	34	34
Female	26	26
BMI	22.77 ± 3.23	22.12 ± 2.12
Pathological stage		
I	27	NA
II	20	NA
III	13	NA
Plaque formation step		
Step 2	12	NA
Step 3	19	NA
Step 4	17	NA
Step 5	14	NA
Hypertension (%)	30 (50.0)	0 (0)***
Smoker	27	23
WBC count, 109/L	6.12 ± 2.07	6.09 ± 2.29
HDL-C, mmol/L	0.93 ± 0.20	1.42 ± 0.31**
LDL-C, mmol/L	2.78 ± 1.02	1.68 ± 0.53**
CR, mmol/L	99.02 ± 11.07	96.923 ± 11.07
TG, mmol/L	1.31 ± 0.33	2.28 ± 0.68*
TC, mmol/L	3.07 ± 1.01	4.53 ± 1.20*
UA, mmol/L	270.99 ± 45.97	270.78 ± 36.93

### 2.2. Plasma and human aortic smooth muscle cells (HAOSMCs)

Fasting blood samples (3 mL) were collected from all participants using EDTA-containing tubes, and then centrifuged at 1200 g for 20 minutes to obtain plasma samples.

HAOSMCs (Cat# 354-05A) were purchased from Sigma-Aldrich were cultured following the manufacturer’s instructions. In brief, cells were rapidly retrieved from liquid nitrogen storage and immediately thawed in a 37°C water bath. Subsequently, the cells were suspended in RPMI-1640 medium, centrifuged at 160 g for 5 minutes, and then resuspended in fresh medium for further experiments. The precipitate was resuspended in 1 mL of RPMI-1640 with 10% fetal bovine serum for cell culture in a 5% CO_2_ incubator with the temperature set at 37°C (Gibco, Rockville, MD). Only 3 to 5 passes of HASMCs were employed in the investigations. The complete in vitro experiment was repeated 3 times.

### 2.3. Cell transfections

pcDNA3.1-MCM3AP-AS1 and -MEF2C vectors were constructed. Mimic of miR-448 and miRNA negative control (NC) were obtained from Sangon Biotech (Shanghai, China). Transfection of 10 nM expression vector or 45 nM miRNA into HAOSMCs was performed using Lipofectamine 2000 (Invitrogen). Empty vector or miRNA NC-transfected cells and un-transfected cells were used as NC and control (C) cells, respectively. For transfection, vectors or miRNA were mixed with Lipofectamine 2000 and incubated with cells for 6 hours. Afterwards, cells were washed with fresh medium, cultured for additional 48 hours and subjected to subsequent experiments.

### 2.4. Dual luciferase activity assay

pGL3-MCM3AP-AS1 wild type (−W) and mutant (−M) luciferase vectors (Promega Corporation) were prepared. Two co-transfection groups were involved in the experiment, including a miR-448 group, consisting of cells co-transfected with miR-448 mimic + MCM3AP-AS1 (W or M) luciferase vector, and a NC group, consisting of cells co-transfected with miRNA NC + MCM3AP-AS1 (W or M) luciferase vector. Luciferase activity was measured at 48 hours of post transfection. All other steps were performed following the manufacturer’s instructions.

### 2.5. RNA preparations

Total RNAs were isolated from cells and plasma samples using RNAzol (Sigma-Aldrich). Samples were mixed with RNAzol to a 10:1 ratio. Then, chloroform was added and mixed with the samples to remove proteins. After centrifugation, the upper liquid layer containing RNA was transferred to new tubes, and ethanol was added to the samples to achieve a final concentration of approximately 75% for total RNA extraction. To harvest miRNA, 85% ethanol was used for RNA precipitation and washing. To precipitate RNA, samples were centrifuged at 13,000 g for 20 minutes at 4°C. DNA removal was performed using DNase I.

### 2.6. RT-qPCR

Following the preparation of cDNA samples, MCM3AP-AS1 and MEF2C mRNA expression was determined by qPCR using All-in-One miRNA qRT-PCR Reagent Kit (Genecopoeia) with GAPDH as the endogenous control, and miR-448 expression was determined with U6 as the endogenous control. Primer sequences were: 5’-GCTGCTAATGGCAACACTGA-3’ (forward) and 5’-AGGTGCTGTCTGGTGGAGAT-3’ (reverse) for MCM3AP-AS1, 5’-CAGGAGGCATTGCTGATGAT-3’ (forward) and 5’-GAAGGCTGGGGCTCATTT-3’ (reverse) for GAPDH; 5’-TCAGGTGACCTCATTTGAACC-3’ (forward) and 5’-GGAGCCATTGCTCATAAGAAAG-3’ (reverse) for MEF2C, and 5’-TTGCATATGTAGGATGTCC-3’ (forward) for miR-448.

### 2.7. Western blot analysis

Isolation of proteins from HAOSMCs was performed using RIPA solution (Invitrogen). Bicinchoninicacid assay (Invitrogen) was performed to measure protein concentration. Denatured protein samples were separated on SDS-PAGE gel (8%). After gel transfer and blocking, the membranes were incubated sequentially with primary antibodies against MEF2C (ab211493, Abcam) and GAPDH (ab9485, Abcam), followed by HRP-conjugated goat anti-rabbit secondary antibody (IgG) (ab97051, Abcam). The protein bands were visualized using ECL (Sigma-Aldrich), and the signal intensity was analyzed using Quantity 1 software (Bio-Rad).

### 2.8. CCK-8 analysis

A 96-well plate was used to culture HAOSMCs (6000 cells per well). CCK-8 solution was added to 10% at 2 hours prior to the measurement of OD values at 450 nm, which was performed every 24 hours for a total of 96 hours.

### 2.9. Statistical analysis

Unpaired t test was used for comparisons of 2 independent groups. ANOVA Tukey’s test was used for comparisons among multiple independent groups. *P* < .05 was considered as statistically significant.

## 3. Results

### 3.1. The expression of MCM3AP-AS1 and miR-448 in atherosclerosis

**Figure 1.**
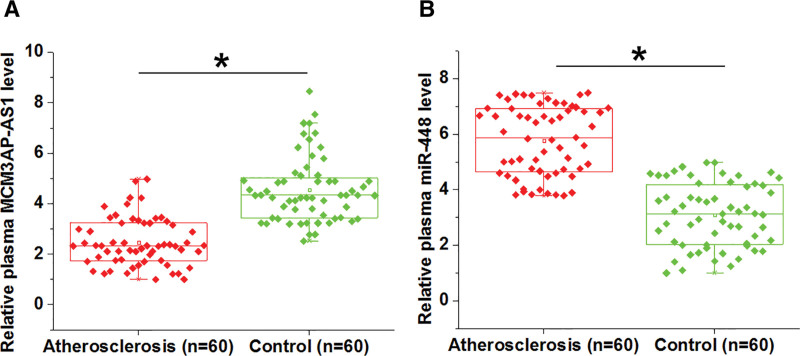
MCM3AP-AS1 was downregulated in atherosclerosis. The expression levels of MCM3AP-AS1 (A) and miR-448 (B) in plasma from atherosclerosis patients (Atherosclerosis group, n = 60) and healthy controls (Control group, n = 60) were measured by RT-qPCR. PCR reactions were repeated 3 times and mean values were compared. *, *P* < .05.

The expression levels of MCM3AP-AS1 (Fig. 1A) and miR-448 (Fig. 4B) in plasma samples of atherosclerosis patients (Atherosclerosis group, n = 60) and healthy controls (Control group, n = 60) were measured by RT-qPCR. Significantly lower expression levels of plasma MCM3AP-AS1 were observed in atherosclerosis group (Fig. 1A, *P* < .05), suggesting that MCM3AP-AS1 may participate in atherosclerosis. Chi-squared t test illustrated that MCM3AP-AS1 was closely correlated with pathological stages and developmental steps (both *P* < .01), but not with patients’ age and gender (both *P* > .05). In contrast, the expression of miR-448 was highly upregulated in atherosclerosis group compared to that in the control group (Fig. 1B, *P* < .05).

**Figure 2.**
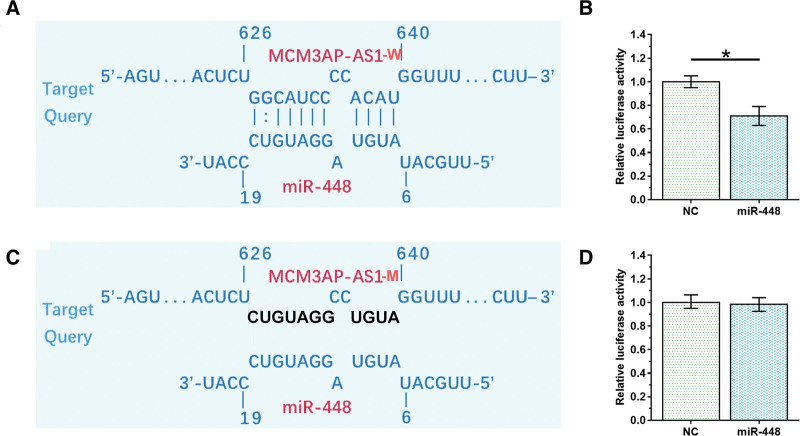
MCM3AP-AS1 and miR-448 interacted with each other. The interaction between MCM3AP-AS1 and miR-448 was analyzed by IntaRNA (http://rna.informatik.uni-freiburg.de/IntaRNA/Input.jsp, (A). Dual luciferase activity assay was performed in 2 co-transfection groups, including miR-448 group (miR-448 mimic + MCM3AP-AS1 (W) luciferase vector) and NC group (miRNA NC + MCM3AP-AS1 (W) luciferase vector). Luciferase activity was measured and compared 48 hours later (B). MCM3AP-AS1 mutant (MCM3AP-AS1-M) was also designed (C) to repeat dual luciferase activity assay (D). NC = negative control.

**Figure 3.**
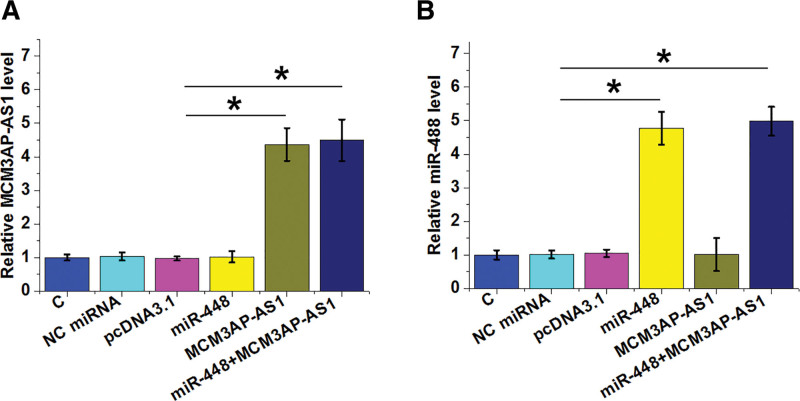
MCM3AP-AS1 and miR-448 did not regulate the expression of each other. HAOSMCs were transfected with either MCM3AP-AS1 expression vector or miR-448 mimic, and the overexpression of MCM3AP-AS1 (A) and miR-448 (B) was confirmed by RT-qPCR. The effects of MCM3AP-AS1 on miR-448 expression (B) and the effects of miR-448 overexpression on MCM3AP-AS1 expression (A) were also analyzed by RT-qPCR. All experiments were repeated 3 times and mean values were presented and compared. *, *P* < .05.

### 3.2. Direct interaction between MCM3AP-AS1 and miR-448 did not affect each other’s expression

IntaRNA prediction revealed that MCM3AP-AS1 and miR-448 could form base pairing (Fig. 2A). To test whether MCM3AP-AS1 and miR-448 can interact with each other, dual luciferase activity assay was performed in 2 co-transfection groups, including a miR-448 group, consisting of cells co-transfected with miR-448 mimic and MCM3AP-AS1 (W or M) luciferase vector, and a NC group, consisting of cells co-transfected with miRNA NC and MCM3AP-AS1 (W or M) luciferase vector. Lower relative luciferase activity was observed in miR-448 group (Fig. 2B, *P* < .05), indicating the direct interaction between them. MCM3AP-AS1 mutant (MCM3AP-AS1-M) was also designed to repeat dual luciferase activity assay (Fig. 2C), while no significant difference in luciferase activity was observed between the 2 groups (Fig. 2D). To further explore the interaction between them, HAOSMCs were transfected with MCM3AP-AS1 expression vector and/or miR-448 mimic, and the overexpression of MCM3AP-AS1 (Fig. 3A) and miR-448 (Fig. 3B) was confirmed by RT-qPCR (*P* < .05). MCM3AP-AS1 did not affect the expression of miR-448 (Fig. 3B) and miR-448 had no effect on the expression of MCM3AP-AS1 (Fig. 3A).

### 3.3. MCM3AP-AS1 overexpression upregulated the expression of MEF2C

**Figure 4.**
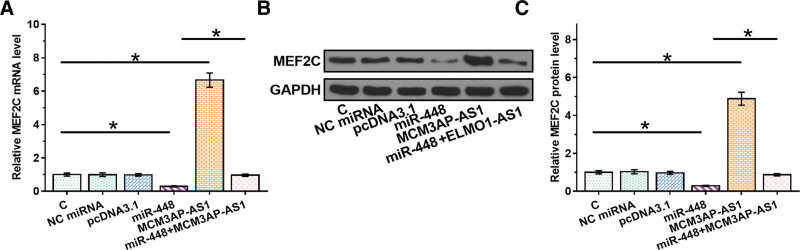
Overexpression of MCM3AP-AS1 increased the expression levels of MEF2C. To test whether MCM3AP-AS1 can serve as the endogenous sponge of miR-448, the effects of overexpression of miR-448 and MCM3AP-AS1 on the expression of MEF2C were analyzed by RT-qPCR (A) and Western blot analysis (B). Western blot data were also quantified (C). All experiments were repeated 3 times and mean values were presented and compared. *, *P* < .05. MEF2 = myocyte enhancer factor 2.

To test whether MCM3AP-AS1 can serve as an endogenous sponge of miR-448, the effects of miR-448 and MCM3AP-AS1 overexpression on the expression of MEF2C were evaluated by RT-qPCR (Fig. 4A) and Western blot analysis (Fig. 4B and C). It showed that overexpression of miR-448 decreased the expression levels of MEF2C (*P* < .05). In contrast, overexpression of MCM3AP-AS1 increased the expression levels of MEF2C and attenuated the effect of miR-448 overexpression (*P* < .05, Fig. 4C).

### 3.4. MCM3AP-AS1 regulated the miR-448/MEF2C axis to inhibit proliferation of HAOSMCs

**Figure 5.**
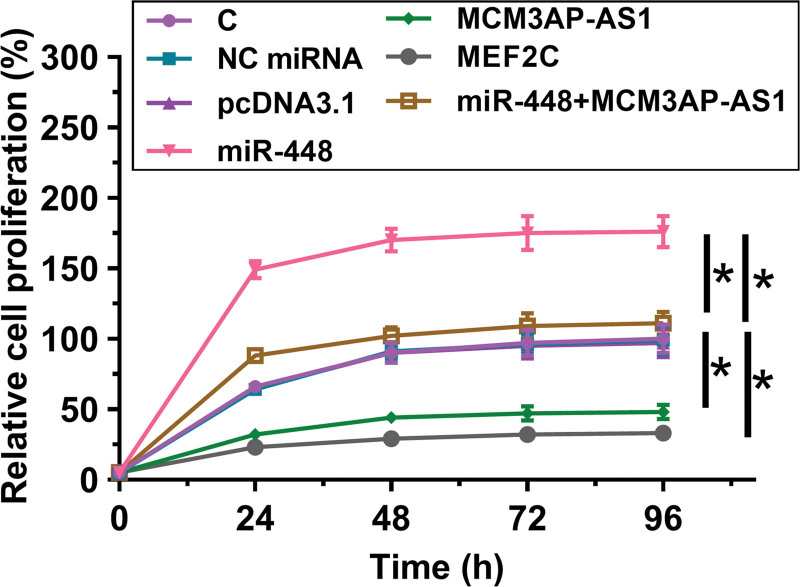
MCM3AP-AS1 regulated the miR-448/MEF2C axis to suppress the proliferation of HAOSMCs. Cell proliferation assay was performed to analyze the effects of MCM3AP-AS1, miR-448 and MEF2C overexpression on the proliferation of HAOSMCs. All experiments were repeated 3 times and mean values were presented and compared. *, *P* < .05. MEF2 = myocyte enhancer factor 2.

Cell proliferation assay was performed to detect the effects of overexpression of MCM3AP-AS1, miR-448 and MEF2C on the proliferation of HAOSMCs (Fig. 5). It showed that overexpression of miR-448 increased cell proliferation rate (*P* < .05), while overexpression of MCM3AP-AS1 and MEF2C played an opposite role (*P* < .05). Moreover, MCM3AP-AS1 suppressed the role of miR-448 in regulating cell proliferation (*P* < .05).

## 4. Discussion

In this study we explored the interactions among MCM3AP-AS1, miR-448 and MEF2C in atherosclerosis. We found that MCM3AP-AS1 was downregulated in plasma of patients with atherosclerosis, and it may sponge miR-448 to upregulate MEF2C in HAOSMCs, thereby suppressing the proliferation of HAOSMCs.

LncRNAs plays critical roles in atherosclerosis.^[17–19]^ For instance, lncRNA ENST00113 activates the PI3K/Akt/mTOR pathway to promote cell proliferation, survival, and migration in atherosclerosis.^[17]^ LncRNA TUG1 regulates ApoM through the miR-92a/FXR1 axis to promote atherosclerosis.^[18]^ LncRNA H19 stimulates the progression of atherosclerosis by regulating the MAPK and NF-kB pathways.^[19]^ MCM3AP-AS1 has been characterized as an oncogenic lncRNA in several types of cancer.^[13–16]^ It is highly expressed in liver cancer and upregulates FOXA1 by sponging miR-194-5p to promote tumor growth.^[13]^ Liang et al^[15]^ reported that MCM3AP-AS1 is overexpressed in papillary thyroid cancer and may promote the invasion and proliferation of cancer cells by sponging miR-211-5p to upregulate SPARC. To our best knowledge, the involvement of MCM3AP-AS1 in other types of human diseases remains unknown. Our study is the first to report the downregulation of MCM3AP-AS1 in atherosclerosis.

Abnormal proliferation of VSMCs plays different roles at different stages of atherosclerosis. At early stage, increased proliferation rate of VSMCs promotes the formation of plaques in arteries, which further promotes development of the disease.^[20]^ In contrast, the promoted proliferation of VSMCs suppresses the rapture of lesions.^[20]^ In this study we showed that overexpression of MCM3AP-AS1 decreases the proliferation rate of HAOSMCs. Therefore, regulating MCM3AP-AS1 expression may contribute to the recovery of atherosclerosis by affecting the proliferation of VSMCs.

A recent study showed that miR-448 could target MEF2C to promote the proliferation of VSMCs.^[12]^ In this study we confirmed the targeting of MEF2C by miR-448 and the enhancing effects of miR-448 on VSMCs proliferation, and further showed that MCM3AP-AS1 and miR-448 could interact with each other, although they did not regulate the expression of each other. In contrast, overexpression of MCM3AP-AS1 attenuates the effects of miR-448 on MEF2C expression and HAOSMCs proliferation. Therefore, we concluded that MCM3AP-AS1 could sponge miR-448 to inhibit its function.

## 5. Conclusion

**Figure 6.**
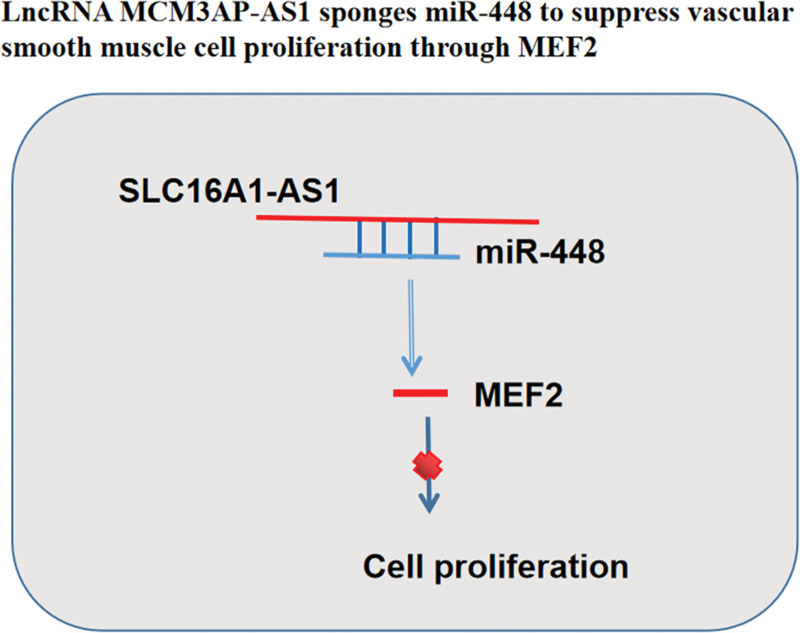
LncRNA MCM3AP-AS1 sponges miR-448 to suppress the proliferation of vascular smooth muscle cell through MEF2. MEF2 = myocyte enhancer factor 2.

MCM3AP-AS1 is downregulated in atherosclerosis and may regulate the miR-448/MEF2C axis to suppress HAOSMCs proliferation (Fig. 6). Although our study provides valuable insights, we acknowledge that the sample size was small, which limits the generalizability of our findings. In addition, the absence of in vivo experiments may have restricted our ability to fully elucidate the mechanisms underlying our observations. Future studies should incorporate a larger sample size and employ animal model experiments to provide more robust evidence for our conclusions.

## Author contributions

**Conceptualization:** Shiping Liu.

**Writing – original draft:** Xiaoyi Qin.
